# Vexed mutations promote degeneration of dopaminergic neurons through excessive activation of the innate immune response

**DOI:** 10.1038/s41531-022-00417-5

**Published:** 2022-11-02

**Authors:** Jacinta Davis, Elizabeth Kolaski, Daniel T. Babcock

**Affiliations:** Department of Biological Sciences, Lehigh University, Bethlehem, PA USA

**Keywords:** Parkinson's disease, Diseases

## Abstract

The hallmark of Parkinson’s disease (PD) is the loss of dopaminergic (DA) neurons in the brain. However, little is known about why DA neurons are selectively vulnerable to PD. We previously completed a screen identifying genes associated with the progressive degeneration of DA neurons. Here we describe the role of a previously uncharacterized gene, *CG42339*, in the loss of DA neurons using *Drosophila Melanogaster*. *CG42339* mutants display a progressive loss of DA neurons and locomotor dysfunction, along with an accumulation of advanced glycation end products (AGEs) in the brain. Based on this phenotype, we refer to *CG42339* as *vexed*. We demonstrate that *vexed* is specifically required within cortex glia to maintain neuronal viability. Loss of *vexed* function results in excessive activation of the innate immune response in the brain, leading to loss of DA neurons. We show that activation of the innate immune response leads to increased nitric oxide signaling and accumulation of AGEs, which ultimately result in neurodegeneration. These results provide further insight into the relationship between the role of the immune response in the central nervous system and how this impacts neuronal viability.

## Introduction

Parkinson’s disease (PD) is the most prevalent movement disorder throughout the world and the second most prevalent neurodegenerative disease^1^. Clinical manifestation of PD includes a wide range of progressive locomotor dysfunctions such as tremors, bradykinesia, and impaired motor coordination^2–4^. This disease is primarily caused by the loss of dopaminergic (DA) neurons located within the substantia nigra^5,6^. There is currently no cure for PD, and the mechanism by which DA neurons are lost remains unknown. With an aging population, the prevalence rate of PD is expected to increase significantly over the next several decades.

Heritable forms of PD are linked to mutations in several genes. The first gene to be associated with PD is α-Synuclein, which acts through a toxic gain-of-function mechanism and is the major component of Lewy Body inclusions^7,8^. Loss of function mutations in Parkin have been associated with PD^9^. Parkin, an E3 ubiquitin ligase, is phosphorylated by PINK1 (PTEN-induced putative kinase 1) to promote mitochondrial quality control through selective mitophagy^10^. Additionally, long-term exposure to pesticides such as rotenone and paraquat that induce oxidative stress are also associated with higher incidence rates of PD^11^. However, identifying what specifically renders DA neurons vulnerable to PD remains unclear. Therefore, finding genes that are involved in the maintenance of DA neurons is crucial in order to discover potential therapeutic targets for PD.

*Drosophila melanogaster* has served as a valuable genetically tractable model system used to help uncover the cellular and molecular mechanisms underlying neurodegenerative diseases such as PD^12^. The Drosophila brain contains ~100 DA neurons organized into distinct clusters that can be easily quantified^13^. These DA neurons in the Drosophila brain are vulnerable to similar factors in PD patients, including expression of α-Synuclein^7,14^, mutations in genes such as PTEN-induced kinase 1 (PINK1) and Parkin^15–17^ and exposure to chemicals such as rotenone^18,19^. Moreover, this loss of DA neurons is linked to locomotor dysfunction which can be similarly recapitulated using Drosophila, further highlighting the utility of Drosophila models of PD^20–22^.

We previously completed a genome-wide screen identifying genes involved in the maintenance of DA neurons in the Drosophila brain^23^. From this screen, we identified the previously uncharacterized gene *CG42339*, which contained single nucleotide polymorphisms (SNPs) that were highly associated with a progressive loss of DA neurons and locomotor dysfunction with age. Although these phenotypes were validated using a mutant allele for CG42339, specific knockdown in DA neurons did not impair DA neuron viability or locomotor activity^23^, suggesting that CG42339 could exert neuroprotective effects in a non-cell autonomous fashion.

Here we describe our results, uncovering the mechanism by which *CG42339* promotes the maintenance of DA neurons. First, we discovered a significant accumulation of advanced glycation end products (AGEs) in the brains of *CG42339* mutants with age, which aligns with the prediction that *CG42339* could be involved in scavenger receptor activity^24^. AGEs are formed when reducing sugars react with free amino groups from proteins. These AGEs will accumulate over time, resulting in tissue damage^25,26^. Previous evidence suggests proteins such as α-Synuclein and tau are glycated in patients with neurodegenerative diseases like Alzheimer’s Disease (AD) and PD^27,28^. Studies regarding PD specifically observed increases in AGE accumulation which was accompanied by increased α-Synuclein aggregation in Lewy bodies^29–31^.

We next demonstrated that *CG42339* is required in cortex glia, as tissue-specific knockdown results in the progressive loss of DA neurons. Since many types of glial cells are associated with the activation of the immune response, we also investigated the potential role of the innate immune response in the phenotypes found in *CG42339* mutants. We found a significant increase in the activity of the innate immune response, as well as increased nitric oxide signaling in *CG42339* mutant brains. It has been suggested that hyperactivation of innate immunity leads to neurodegeneration^32,33^. Brain inflammation in the central nervous system (CNS) can escalate the production of reactive oxygen species and reactive nitrogen species, which is linked to neurodegeneration, specifically PD^34,35^.

Finally, we demonstrated that both AGE accumulation and increased nitric oxide signaling are downstream of innate immune response activation. Altogether, these results further highlight the complex relationship between the immune response and the central nervous system and how these links can impact neurodegeneration.

## Results

### Neurodegeneration and locomotor dysfunction in *CG42339* mutants

**Fig. 1:**
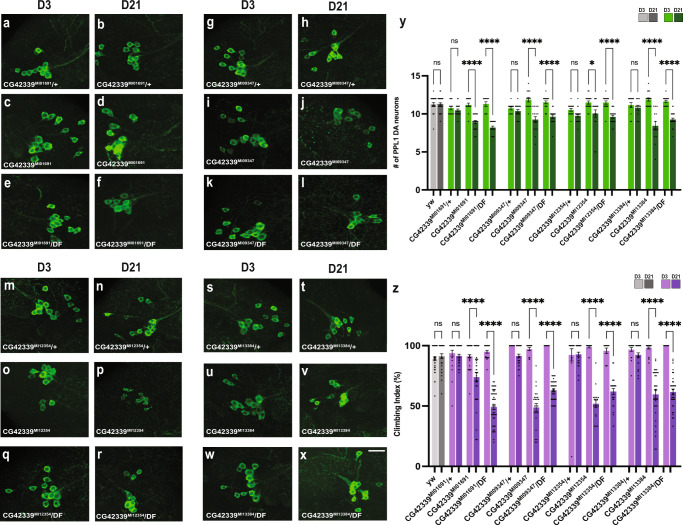
Progressive loss of DA neurons and locomotor defects in CG42339 alleles. **A**–**Y** Progressive loss of PPL1 neurons stained with anti-tyrosine hydroxylase (green). Neuronal loss was assessed in *CG42339*^*MI01691*^ (**A**–**F**), *CG42339*^*MI09347*^(**G**–**L**), *CG42339*^*MI12354*^ (**M**–**R**), and *CG42339*^*MI13384*^ (**S**–**X**). Each allele was assessed in both heterozygous and homozygous conditions, as well as over the deficiency *BSC540* that spans this region. Images were taken at 20x magnification with Z stack slice interval of 1.00 μm zoomed to 3.5x. **Z** Average climbing ability was measured for each allele on Day 3 and Day 21. Individual data points are shown with black dots. Error bars represent the SEM. ****p < 0.0001; *p < 0.05; n.s. not significant using Brown–Forsythe and Welch ANOVA tests with post hoc Games–Howell’s multiple comparisons. Scale bar in **X** is 20 μm for **A**–**X**.

We previously identified *CG42339* in a screen for genes associated with progressive degeneration of DA neurons^23^. To better understand the role of this previously uncharacterized gene, we expanded our analysis to all publicly available *CG42339* mutant alleles. While homozygous mutants for each of these alleles displayed a normal number of DA neurons in the protocerebral posterior lateral-1 (PPL1) cluster on Day 3, they each showed a significant decrease in PPL1 neurons by Day 21 relative to heterozygous controls (Fig. 1A–Y). To confirm that this progressive loss of DA neurons is due to the loss of *CG42339* function, we also measured neuron viability in mutant alleles crossed to a deficiency that spans the *CG42339* region. We found a similar loss of DA neurons in each condition compared to homozygous mutants, suggesting that disruption of *CG42339* function results in progressive degeneration of DA neurons (Fig. 1A–Y).

While our initial measurements focused on PPL1 neurons, we also assessed the neuronal viability of other DA neuron clusters in the Drosophila brain. We also found a progressive, age-dependent loss of DA neurons located in the protocerebral posterior medial 1/2 (PPM1/2) cluster (Supplementary Fig. 1), demonstrating that the role of *CG42339* in maintaining DA neurons is not limited to one subset of neurons. Interestingly, mutations in *CG42339* did not result in the loss of DA neurons located within the Protocerebral Posterior Medial 3 (PPM3) cluster (Supplementary Fig. 2), highlighting that not all DA neurons are similarly vulnerable to specific mutations.

Since the loss of DA neurons is often tied to locomotor dysfunction^12,21^, we also assessed the climbing ability in *CG42339* mutants. Heterozygous controls for each mutant allele displayed normal locomotor activity at both Day 3 and Day 21, while homozygous flies showed a progressive loss of motor activity by Day 21 (Fig. 1Z). Similar to our findings with DA neuron viability, flies bearing a mutation along with the corresponding deficiency also showed a loss of motor activity (Fig. 1Z). Together, these results demonstrate that *CG42339* is required to maintain DA neuron viability and locomotor activity with age.

### Accumulation of advanced glycation end products in *CG42339* mutant brains

Although no previous experimental data has been published for these alleles, sequence analysis suggests that *CG42339* may be associated with scavenger receptor activity^24^. Scavenger receptors target a wide range of molecules, including bacteria, cellular debris, and advanced glycation end products (AGEs), and these receptors are often closely linked with the innate immune response^36^.

**Fig. 2:**
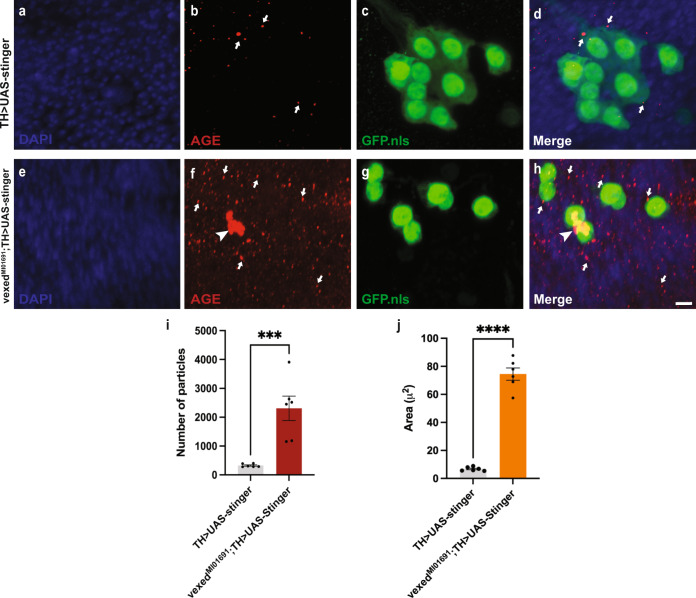
Accumulation of advanced glycation end products in CG42339 mutant brains. **A–H** AGE accumulation in flies at Day 21 with DA-neuron specific expression of a nuclear-localized GFP (*UAS-Stinger*) in a wildtype (**A**–**D**) or *CG42339* mutant (**E**–**H**) background. Arrows designate small puncta of AGEs observed throughout the brain. Arrowheads designate large aggregates of AGEs found within close proximity to PPL1 neurons. Images were taken at 63x magnification with a Z stack slice interval of 0.88 μm zoomed to 3.5x. **I** Average number of AGE particles found within an area of 1500 μm^2^ in both wildtype and *CG42339* mutant brains. **J** Average area occupied by AGE staining within an area of 1500 μm^2^ in both wildtype and *CG42339* mutant brains. Individual data points are shown with black dots. Error bars represent the SEM. ****p < 0.0001; ***p < 0.001; using a Student’s *t*-test. Scale bar in **H** is 5 μm for **A**–**H**.

AGEs are formed by the excessive glycation of proteins, lipids, and nucleic acids, and the accumulation of AGEs is associated with several age-related diseases. Long-lived proteins, in general, are susceptible to glycation, including several proteins associated with neurodegenerative diseases^27^. To assess the potential role of *CG42339* as a scavenger receptor, we measured the accumulation of AGEs in aged wild-type brains in comparison to those of *CG42339* mutants. While there was only a sparse accumulation of AGEs in wild-type brains at Day 21 (Fig. 2A–D), we noted a widespread accumulation of AGEs in *CG42339* mutants (Fig. 2E, F). Since much of this staining was found in regions near clusters of DA neurons, we also labeled these neurons with a nuclear-localized Green Fluorescent Protein (*UAS-Stinger)*^37^, to determine their proximity to one another. We found a significant accumulation of AGEs in the vicinity of DA neurons within the PPL1 cluster, including large particles that appear to have aggregated in this region (Fig. 2H–J). Interestingly, we found no significant accumulation of AGEs in the region of the brain surrounding PPM3 in vexed mutants compared to controls (Supplementary Fig. 3). This could explain why PPM3 neurons are not lost in vexed mutant brains. Together, these results demonstrate that *CG42339* is required to promote the turnover of AGEs and prevent their accumulation in the nervous system. Since this phenotype of accumulated AGEs in *CG42339* mutants is similar to that shown for the human receptor for advanced glycation end products (RAGE)^38,39^, we refer to *CG42339* as *vexed* (*vex*).

### Vexed is required in glial cells to maintain DA neurons and locomotor function

**Fig. 3:**
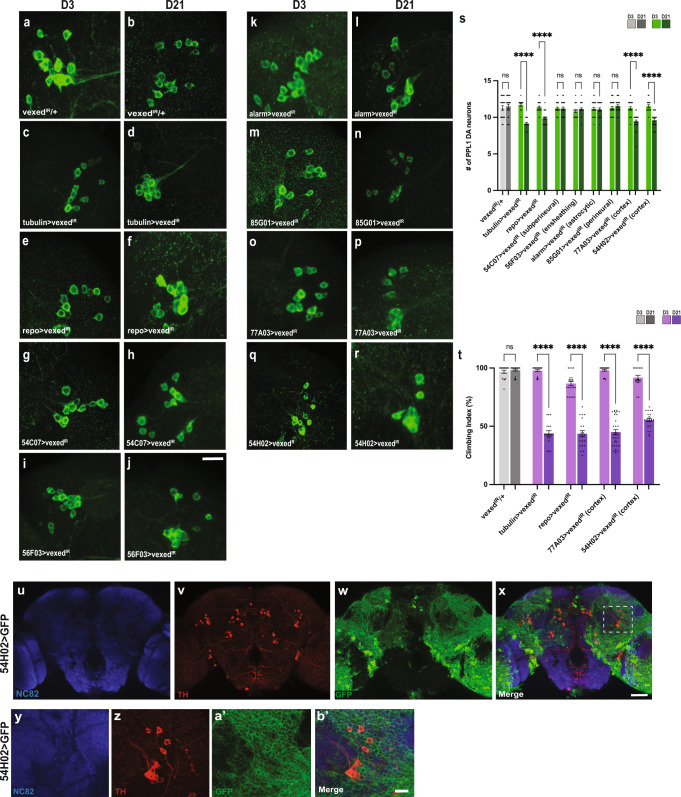
Vexed expression is required in cortex glia. **A–S** Clusters of PPL1 neurons stained with anti-tyrosine hydroxylase (green) upon tissue-specific knockdown of vexed. Comparison of controls (**A**, **B**) to ubiquitous knockdown (**C**, **D**), and knockdown in all glia (**E**, **F**), subperineural glia (**G**, **H**), ensheathing glia (**I**, **J**), astrocytic glia (**K**, **L**), perineural glia (**M**, **N**), and cortex glia (**O**–**R**). Images were taken at 20x magnification with a Z stack slice interval of 1.00 μm zoomed to 3.5x. **T** Average climbing ability measured for each condition at Day 3 and Day 21. **U**–**X** Anatomical organization of cortex glia (green) surrounding the DA neurons (red). **Y**–**B**′ Higher magnification of images panels **U**–**X** highlighting cortex glia surrounding DA neurons in the PPL1 cluster. Images were taken with Z stack slice intervals set at 1.00 μm. Individual data points are shown with black dots. Error bars represent the SEM. ****p < 0.0001 using Brown–Forsythe and Welch ANOVA tests with post hoc Games–Howell’s multiple comparisons. Scale bar in **J** is 20 μm for **A**–**R**. Scale bar in **X** is 40 μm. Scale bar in **B**′ is 20 μm.

To discover the tissue(s) in which *vexed* function is required to maintain DA neurons, we used the Gal4/UAS system to perform tissue-specific knockdown of *vexed* using RNAi^40^. We previously showed that the knockdown of vexed in DA neurons did not result in neuronal loss^23^, suggesting that Vexed could be working in a non-cell autonomous fashion to protect these neurons. We first used tubulin-gal4^41^ to knock down *vexed* ubiquitously and found a significant loss of DA neurons in both the PPL1 and PPM1/2 neurons (Fig. 3D and S; Supplementary Fig. 4). Similarly, to our results with *vexed* mutants, there was no loss of PPM3 neurons (Supplementary Fig. 5). Since ubiquitous knockdown of *vexed* shares, a similar phenotype to mutants, we next sought to identify specific cell types where Vexed is required. We hypothesized that Vexed could provide non-cell autonomous protection to neurons by acting in glial cells. When *vexed* was knocked down in all glia using repo-gal4^42^, we found DA neuron loss at Day 21 (Fig. 3F, S), demonstrating that *vexed* is required in glial cells.

Glial cells carry out a number of vital functions in the Drosophila nervous system, many of which are analogous to those of mammalian glial cells^43–46^. To determine the type(s) of glial cells in which Vexed is required, we next knocked down expression in individual glial cell subtypes using specific Gal4 drivers. DA neuron viability was assessed using RNAi in perineural glia (85G01-Gal4)^47^, subperineural glia (54C07-Gal4)^47^, ensheathing glia (56F03-Gal4)^47^, astrocytic glia (alarm-Gal4)^48^, and cortex glia (77A03-Gal4)^47^ and (54H02-Gal4)^47^ (Fig. 3G–S). There was no progressive degeneration of DA neurons in the PPL1 and PPM1/2 clusters when vexed was knocked down in perineural, subperineural, ensheathing, or astrocytic glia. However, we did find a progressive loss of DA neurons upon knockdown of vexed in cortex glia using two independent drivers (Fig. 3O–S and Supplementary Fig. 3), highlighting the need for Vexed function within these cells to maintain neuronal viability. Knocking down *vexed* using the ubiquitous, pan-glia, or cortex glia drivers also resulted in a progressive loss of locomotor function by Day 21 (Fig. 3T), again recapitulating the mutant phenotypes.

Cortex glia contains processes that surround neuronal cell bodies all throughout the protocerebrum in the Drosophila brain^49^, including the areas where the clusters of dopamine neurons are located (Fig. 3U–B′). Together, these results highlight the neuroprotective role of vexed in cortex glia.

### Neurodegeneration in vexed mutants is caused by the activation of the innate immune response

**Fig. 4:**
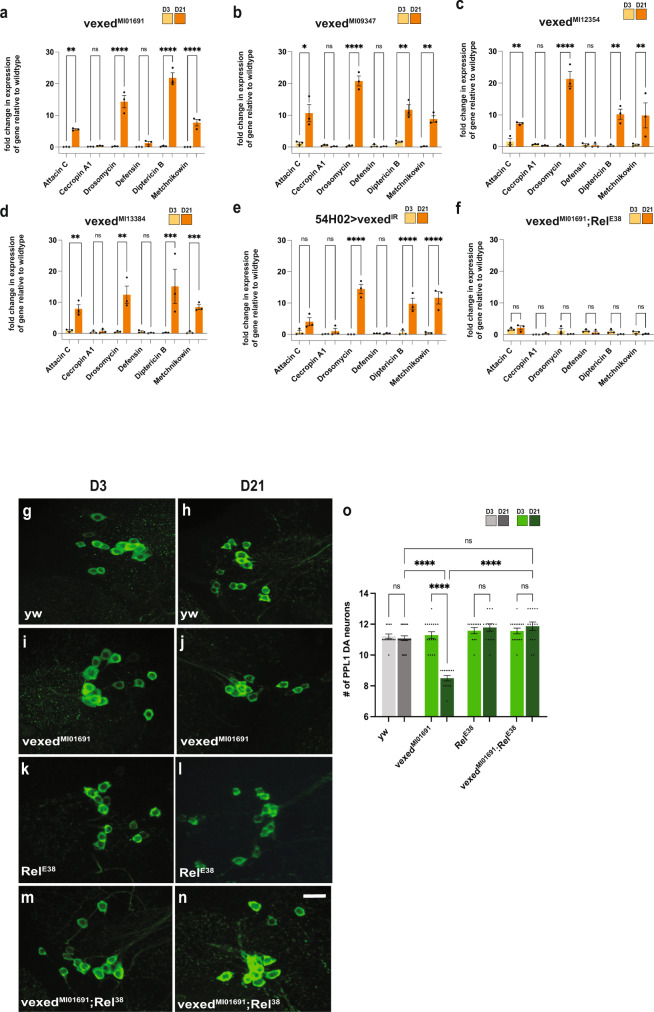
Loss of DA neurons in vexed mutants is caused by activation of the innate immune response. **A–F** qPCR assessing transcript levels of several antimicrobial peptides from heads of *vexed* mutant alleles (**A**–**D**), RNAi-mediated knockdown of *vexed* in cortex glia (**E**), and flies mutant for both *vexed* and *relish* (**F**). **G**–**O** Measurement of PPL1 neuron viability in wildtype controls (**G**, **H**), *vexed* mutants (**I**, **J**), *relish* mutants (**K**, **L**), and flies with mutations in both *vexed* and *relish* (**M**, **N**). Images were taken at 20x magnification with a Z stack slice interval of 1.00 μm zoomed to 3.5x. Individual data points in each graph are shown with black dots. Error bars represent the SEM. ****p < 0.0001; ***p < 0.001; **p < 0.01; *p < 0.05; n.s. not significant using Brown–Forsythe and Welch ANOVA tests with post hoc Games–Howell’s multiple comparisons. Scale bar in **N** is 20 μm for **G**–**N**.

The relationship between the nervous system and the innate immune system has received growing attention recently, particularly in the context of neurodegenerative diseases. While the link between the innate immune response and neurodegeneration has been documented, it was only more recently determined that excessive activation of the innate immune response can itself result in neuronal loss^50^. As both glia activation and the accumulation of AGEs are linked to the innate immune response, we measured innate immune response activity in *vexed* mutant alleles as well as with RNAi knockdown in cortex glia. To assess activation of the innate immune response, we measured the expression levels of several antimicrobial peptides (AMPs) as a readout^51–53^. *Vexed* mutants displayed a significant increase in the levels of Attacin C, Drosomycin, Diptericin B, and Metchnikowin across all allelic variants at day 21 (Fig. 4A–D)^54^. In contrast, heterozygous *vexed* mutants that do not lose DA neurons showed no significant increase in AMP expression (Supplementary Fig. 6). Upon knockdown of *vexed* in the cortex glia, we tested the identical AMPs and found that there was an increase in expression for Drosomycin, Diptericin B, and Metchnikowin (Fig. 4E). These results demonstrate that the loss of *vexed* function results in a significant increase in innate immune activity in the nervous system. AMP expression is primarily determined by the activation of well-characterized transcription factors, including *relish*^55,56^. To determine whether the increase in AMP expression in *vexed* mutants is mediated by *relish*, we examined AMP levels in flies mutant for both *vexed* and *relish*. We found that double mutants did not show a significant increase in AMP expression at Day 21 (Fig. 4F), suggesting that the innate immune activity in *vexed* mutants requires *relish* activity.

We next asked if the excessive innate immune response is responsible for the loss of DA neurons in *vexed* mutants. Consistent with our previous results, *vexed* mutants showed a progressive loss of PPL1 and PPM1/2 DA neurons by Day 21. However, *relish* mutants alone did not have a significant loss of neurons in these clusters. Interestingly, the loss of DA neurons from *vexed* mutations is prevented by introducing the *relish* mutation (Fig. 4G–O and Supplementary Fig. 7). Together, these results demonstrate that degeneration of DA neurons in *vexed* mutant brains is due to excessive activation of the innate immune response.

### Enhanced nitric oxide signaling in *vexed* mutant brains

**Fig. 5:**
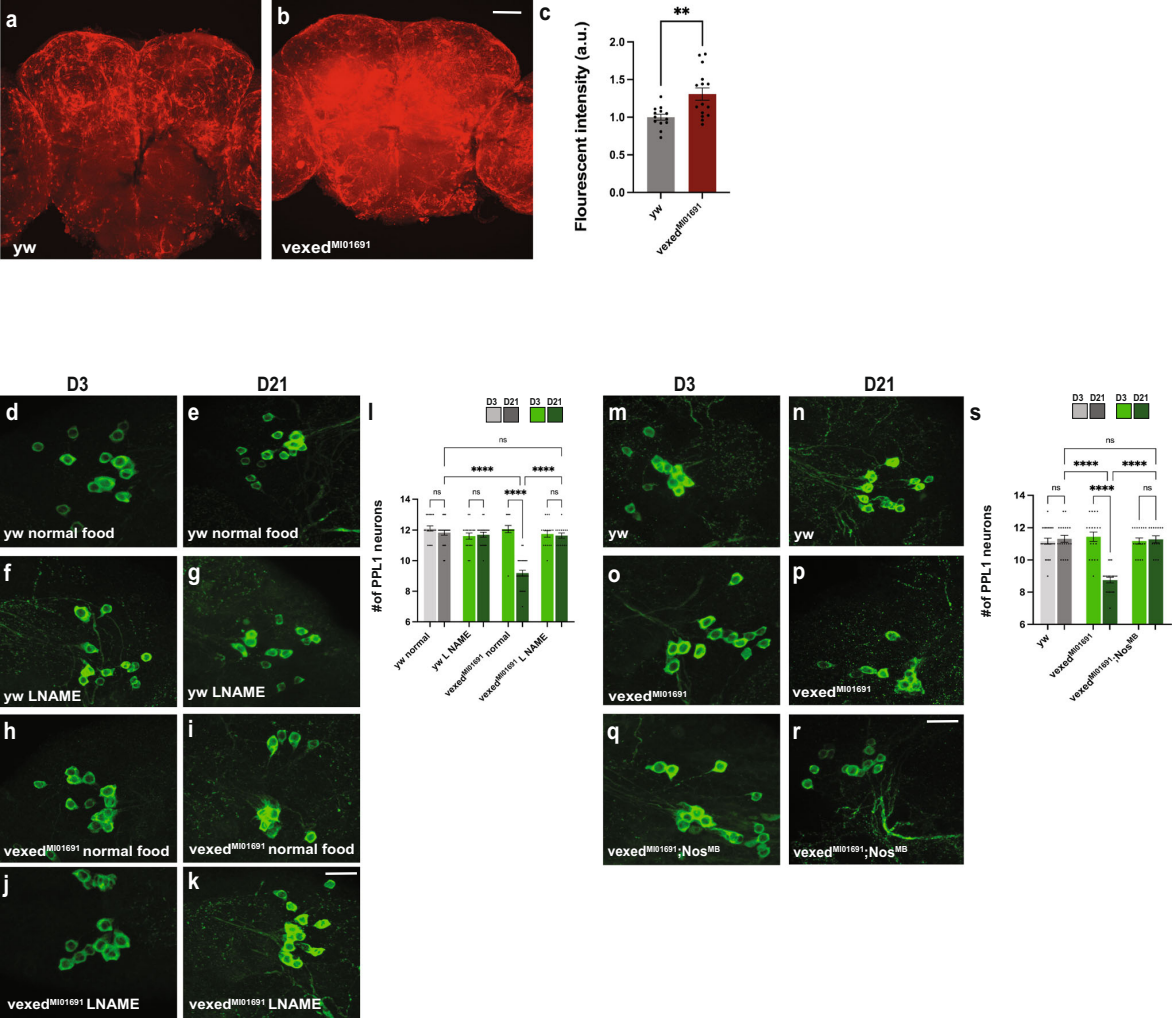
Limiting nitric oxide signaling rescues DA neuron loss in vexed mutant brains. **A**–**C** Measurement of fluorescent intensity of DAR 4 M AM dye in wildtype (A) and vexed mutant (B) brains. **D**–**L** Viability of PPL1 neurons upon pharmacological inhibiting nitric oxide signaling with L-NAME. Conditions tested were wild-type controls raised on standard (**D**, **E**) or L-NAME-supplemented food (**F**, **G**) and vexed mutants raised on standard (**H**, **I**) or L-NAME-supplemented food (**J**, **K**). **M**–**S** Viability of PPL1 neurons upon genetically inhibiting nitric oxide signaling using a *nos* mutant allele. Conditions tested were wild-type controls (**M**, **N**), *vexed* mutants (**O**, **P**), and flies with mutations in both *vexed* and *nos* (**Q**, **R**). Images were taken at 20x magnification with a Z stack slice interval of 1.00 μm zoomed to 3.5x for (**D**–**K**) and (**M**–**R**). No further magnification (**A**, **B**). Individual data points in each graph are shown with black dots. Error bars represent the SEM. ****p < 0.0001; **p < 0.01; n.s. not significant using a Student *t*-test in **C** and a Brown–Forsythe and Welch ANOVA tests with post hoc Games–Howell’s multiple comparisons in **L** and **S**. Scale bar in **B** is 40 μm for **A**, **B**. Scale bar in **K** is 20 μm for **D**–**K**. Scale bar in **R** is 20 μm for **M**–**R**.

To better understand the mechanism responsible for the degeneration of DA neurons in *vexed* mutants, we next expanded our investigation to include signaling pathways that are associated with the accumulation of AGEs and innate immune activity. As nitric oxide activity is associated with these factors, we next measured nitric oxide activity in both wildtype and *vexed* mutant brains using DAR 4 M AM dye as a fluorescent nitric oxide indicator^57^. We found a significant increase in fluorescent signaling in aged *vexed* mutants compared to wild-type controls (Fig. 5A–C), showing that nitric oxide is enhanced in *vexed* mutants.

If enhanced nitric oxide signaling is associated with the loss of DA neurons in *vexed* mutants, we hypothesized that reducing nitric oxide signaling would be neuroprotective. To limit nitric oxide signaling pharmacologically, both wildtype and *vexed* mutants were raised on food supplemented with the nitric oxide inhibitor l-Name (l-nitro arginine methyl ester)^58^ or standard food. As shown previously, *vexed* mutants raised on standard food show a progressive loss of DA neurons (Fig. 5D–L and Supplementary Fig. 8). However, *vexed* mutants raised on l-NAME food maintain DA neurons in these clusters, demonstrating the neuroprotective effect of reducing nitric oxide signaling.

We also reduced nitric oxide signaling genetically using a mutation in nitric oxide synthase (nos) (*Nos*^*MB04018*^)^59^. Similar to our results with l-NAME, we found that double mutants for *vexed* and *nos* maintained DA neurons at Day 21 (Fig. 5M–S). Together, these results demonstrate that the loss of DA neurons in *vexed* mutants is due to increased nitric oxide signaling, and that reducing nitric oxide signaling has neuroprotective effects.

### Nitric oxide and AGE accumulation in *vexed* mutants depends on enhanced immune activity

We demonstrated that *vexed* mutants showing progressive loss of DA neurons and locomotor function also exhibit accumulation of AGEs, activation of the innate immune response, and nitric oxide signaling. To better understand these phenotypes and how they impact neurodegeneration, we next investigated any possible causal relationships between them.

**Fig. 6:**
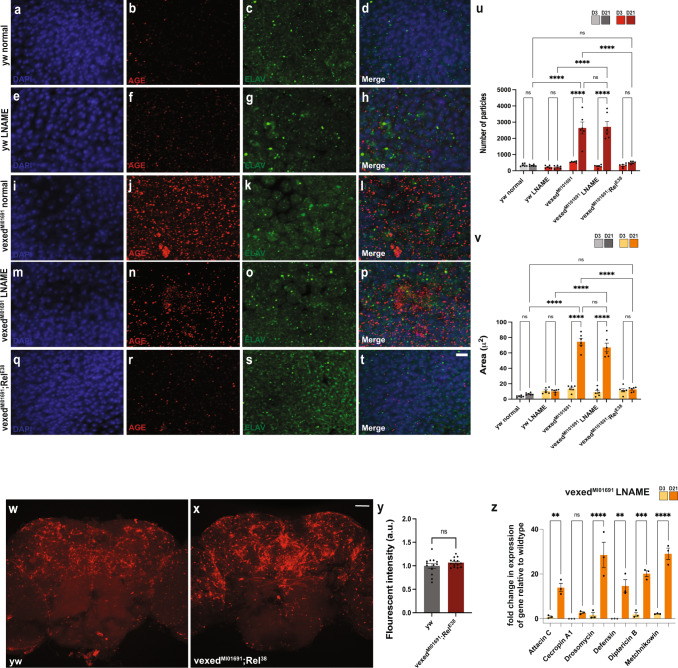
AGE accumulation and nitric oxide signaling in vexed mutants is downstream of the innate immune response. **A**–**V** Measurement of AGE accumulation in brains of wild-type flies raised on standard (**A**–**D**) or L-NAME-supplemented food (**E**–**H**), vexed mutant flies raised on standard (**I**–**L**) or L-NAME-supplemented food (**M**–**P**), and flies with mutations in both vexed and relish (**Q**–**T**). **U** Average number of AGE particles found within an area of 1500 μm^2^ in each condition. **V** Average area occupied by AGE staining within an area of 1500 μm^2^ in each condition. **W**–**Y** Fluorescent intensity of DAR 4 M AM dye in wild-type brains compared to flies with mutations in both vexed and relish. **Z** qPCR assessing transcript levels of antimicrobial peptides from heads of *vexed* mutants raised on L-NAME-supplemented food. Images were taken at 63x magnification with Z stack slice interval of 0.88 μm zoomed to 3.5x for (**A**–**T**). Images were taken at 20x magnification with a Z stack slice interval of 1.00 μm (**W**–**X**). Individual data points in each graph are shown with black dots. Error bars represent the SEM. ****p < 0.0001; ***p < 0.001, **p < 0.01; n.s. not significant using a Student *t*-test for **Y** and a Brown–Forsythe and Welch ANOVA tests with post hoc Games–Howell’s multiple comparisons for **U**, **V**, and **Z**. Scale bar in **T** is 4 μm for **A**–**T**. Scale bar in **X** is 40 μm for **W**, **X**.

To test the relationship between AGE accumulation and nitric oxide signaling, we measured AGE accumulation in the brains of both wildtype and *vexed* mutants that were raised on either standard media or media supplemented with l-NAME. If AGE accumulation depends on nitric oxide signaling, then we would expect the food supplemented with l-NAME to reduce AGE accumulation in *vexed* mutants. However, we found that raising *vexed* mutants on media supplemented with l-NAME had no impact on AGE accumulation, as the amount of AGEs found in *vexed* mutant brains was no difference between normal and l-NAME media (Fig. 6M–P, U, V). These results suggest that AGE accumulation is not a downstream consequence of nitric oxide signaling.

We next assessed the relationship between AGE accumulation and activation of the innate immune response. If AGE accumulation depends on innate immune activation, then we would expect reduced AGE burden in the brains of flies mutant for both *vexed* and *relish*. We found that these double mutants showed a significant decrease in AGE burden compared to *vexed* mutants alone (Fig. 6Q–V), demonstrating that AGE accumulation in *vexed* mutants requires activation of the innate immune response.

The relationship between nitric oxide signaling and the innate immune response was assessed by measuring the intensity of DAR 4 M AM dye staining in the brains of flies mutant for both *vexed* and *relish*. If the enhanced nitric oxide signaling found in vexed mutants depends on the activation of the innate immune response, we would also expect these double mutants to have a similar dye intensity to controls. Indeed, we found that these double mutants no longer displayed an increase in nitric oxide signaling (Fig. 6W–Y), suggesting that nitric oxide signaling in vexed mutants also requires activation of the innate immune response.

Finally, we asked whether nitric oxide signaling is required for the activation of the innate immune response in vexed mutants. If enhanced nitric oxide signaling is required to activate the innate immune response, then we would expect *vexed* mutants raised on media supplemented with l-NAME to have reduced levels of AMP transcription. However, we found that AMP expression remained elevated when flies were raised on L-NAME food (Fig. 6Z). Altogether, these results demonstrate that activation of the innate immune response is required for both the accumulation of AGEs and enhanced nitric oxide signaling in vexed mutant brains.

## Discussion

In this current study, we characterized the role of the previously unannotated gene vexed in the non-cell autonomous maintenance of DA neurons through the regulation of the innate immune response. We also found that DA neuron loss and locomotor dysfunction is accompanied by AGE accumulation and nitric oxide signaling in vexed mutants. Finally, we found that Inhibiting innate immunity led to DA neuron rescue, decreased AGE accumulation, and reduced nitric oxide signaling, suggesting that *vexed* mutations contribute to the hyperactivity of innate immunity, which stimulates AGE and nitric oxide accumulation.

The observation that Vexed exerts neuroprotective effects non-cell autonomously in cortex glia further highlights the importance of glia in the pathogenesis of neurodegenerative diseases. Recent investigations of glia in PD have garnered substantial attention. α-Synuclein is a protein that promotes synaptic vesicle trafficking, but when mutated, the protein will misfold, forming aggregates and bringing about the death of DA neurons^60,61^. Studies have suggested that the overactivation of glia promotes an increase in the aggregate formation of α-Synuclein^62,63^. Based on our findings and previous evidence, *vexed* may function in a similar manner or may enhance glia activation and impair other cellular processes.

Scavenger receptors perform a wide range of functions, including the removal of apoptotic cells and maintaining homeostasis by lipid transport. These receptors also clear the products of oxidative stress^64,65^. Our data and previous bioinformatic data suggest that Vexed likely acts as a scavenger receptor. Along with our results demonstrating that knockdown of Vexed in cortex glia is sufficient to induce activation of the innate immune response (Fig. 4), this suggests that defective scavenger activity of cortex glia induces the innate immune response. This idea is consistent with the observation that Advanced Glycation End Products (AGEs) are among common targets for scavenger receptors^66^ and the accumulation of AGEs we find in vexed mutant brains (Fig. 2).

Among the most well-studied scavenger receptors in Drosophila is Draper, which stimulates glial phagocytosis and clears apoptotic cells^67–70^. Evidence suggests that the upregulation of *draper* promotes the loss of neurons as well as locomotor dysfunction^68^. Specifically, *draper* was found to be expressed in cortex glia and cleared debris from dead neurons^71^. It will be interesting to examine in future studies whether Vexed may prevent AGE accumulation and oxidative stress normally through the cortex glia similarly to how Draper works.

Glial cells are the predominant phagocytes in the Drosophila central nervous system^49^. While this is most commonly associated with ensheathing glia^48^, cortex glia have been shown to phagocytose dead neurons in the developing optic lobe^71^.

The results from this study also highlight differences in AGE accumulation in various regions of the brain. While AGE accumulation is most prominent in regions surrounding DA neurons that are lost in *vexed* mutants, we did not find this accumulation in areas surrounding PPM3 neurons. Perhaps the anatomical location of these neurons renders them more resistant to this pathology. Although cortex glia clearly associates with DA neurons located within the PPL1 and PPM3 clusters (Fig. 3X), it is unclear whether cortex glia throughout the brain all react in an identical manner. Additionally, the use of fluorescent AMP reporters in previous studies did not show a uniform expression pattern in the central brain^50^. Differences in the induction of the innate immune response between regions of the brain could also explain differences in vulnerability.

Finally, it will also be interesting to examine the human ortholog of Vexed, Somatomedin-B, and thrombospondin type-1 domain-containing protein (SBS-PON). While most of the data regarding this gene comes from bioinformatic analysis^72–74^, it will also be of great interest to determine whether the neuroprotective effects found in our current study are conserved.

## Methods

### Fly stocks and husbandry

*Drosophila melanogaster* stocks were maintained at 25 °C on standard Drosophila media. Flies were collected at eclosion, separated by sex, and aged for either 3 days or 21 days at 29 °C. During the aging experiments, the flies were transferred to new media every 3 days. The following stocks were obtained from the Bloomington Drosophila Stock Center: CG42339^MI01691^ (#32773)^75^, CG42339^MI13384^ (#59652)^75^, CG42339^MI12354^ (#57935)^75^, CG42339^MI09347^ (#51278)^75^, UAS-CG42339^IR^ (#67273)^76^, Relish^E38^ (#9458)^77^, Df BSC540 (#25068)^78^, y[1]w[1] (#1495)^79^, tubulin-Gal4 (#5138)^41^, repo-Gal4 (#7415)^42^, 85G01-Gal4 (#40436)^47^, 56F03-Gal4 (#39157)^47^, 54C07-Gal4 (#50472)^47^, alarm-Gal4 (#67032)^48^, 77A03-Gal4 (#39944)^47^, 54H02-Gal4 (#45784)^47^, UAS-Stinger (#84277)^37^, Nos^MB04018^ (#24283)^59^.

### Immunohistochemistry

Brains were dissected and stained as described previously^23^. Brains were dissected in 1X PBS and fixed for 20 min in 4% paraformaldehyde at room temperature. Brains were then washed four times using PBS with 0.3% Triton x-100 (0.3% PBST) for five minutes each. Samples were then placed in blocking buffer (PBS with 0.2% Triton x-100 and 0.1% normal goat serum) for a minimum of 1 h at 4 °C. After incubating with the blocking buffer, primary antibody was added and left on the sample for 48 h at 4 °C. Samples were then washed with 0.3% PBST four times for 5 min each at room temperature. Secondary antibodies were then added and samples were left to incubate at 2 h in the dark at room temperature. Samples were then washed with 0.3% PBST four times at 5 min each. Brains were then mounted using Vectashield mounting media (Vector Laboratories). Slides were imaged and then preserved at −20 °C. Primary antibodies used include rabbit anti-tyrosine hydroxylase (1:100, AB152, Millipore), rabbit anti-advanced glycation end products (1:100, ab23722, Abcam), rat anti-elav (1:20, Developmental Studies Hybridoma Bank), and chicken and-GFP (1:500, A10262, Thermo Fisher). Secondary antibodies used: Alexa Fluor 488 goat anti-rabbit, 568 goat anti-rabbit, 488 goat anti-rat (1:200, Fisher Scientific), and DAPI (1:1000).

### Dopaminergic neuron quantification

DA neurons were counted using a Nikon Eclipse Ni-U fluorescent microscope equipped with a 20x objective. DA neurons of interest located in the protocerebral posterior lateral-1 (PPL1), Protocerebral posterior medial 1 and 2 (PPM1/2), and Protocerebral posterior medial 3 (PPM3) clusters were counted for both hemispheres within each individual brain sample. Brains were analyzed by genotype and sex for each experiment. A minimum of ten brains were analyzed for each group. Male and female brains were analyzed separately and then combined if there was no statistical difference between them. For mutants of *CG42339* located on the X chromosome, only hemizygous males were analyzed. Experiments were performed in triplicate and were scored blindly with regard to genotype and condition.

### Quantification of advanced glycation end products

AGE accumulation in brain samples was quantified using the Analyze Particles tool in FIJI (FIJI Is Just ImageJ)^80^. Z stacks of 48 slices (Interval of 0.88 μm) of the AGEs accumulating around the DA neurons were obtained and processed. The background was subtracted and the threshold was adjusted uniformly across all images. The total amount of particles and area were quantified over 6 images for each condition on both day 3 and day 21.

### Image analysis

Microscopic images were taken using a Zeiss LSM 880 confocal microscope. A 20x objective was used to image neuronal clusters in each brain sample. AGE staining images were obtained using a 63x oil objective. All images, unless stated otherwise in the figure legend, are zoomed in to 3.5x. Confocal stacks were generated using parameters specifically outlined under each assay description in the figure legends. Brightness and contrast were then adjusted using FIJI (FIJI Is Just ImageJ)^80^ and Adobe Photoshop CC2020. All images were processed using the same parameters for brightness and contrast specific to each data set. Figures were then assembled in Adobe Illustrator CC2020.

### Locomotor behavior

Flies were collected shortly after eclosion and separated by sex. Groups consisted of 10 male or female flies in each vial, with a total of 80–100 flies per genotype. Hemizygous males only were used for X chromosomal mutations. All other groups were combined since we saw no differences between males and females. We performed these experiments when the flies were aged to day 3 and day 21 at 29 °C. Flies were transferred onto new food every 3 days. We begin the climbing assay by transferring each group of flies into a tube that is made up of two glass vials connected at the open ends (total diameter, 2.5 cm; total height, 20 cm). Each group acclimated in the glass tube for 5 min. The climbing index is measured for each group by the percentage of flies that are able to climb to an 8 cm mark indicated on the glass vials in 20 s. The timer for these trials begins when the glass vial is tapped down onto a mouse pad. Three trials were carried out for each group of flies. After each trial, the flies were allowed 1 min to recover.

### *N*-nitro-l-arginine methyl ester supplement

Standard fly media was prepared with 50 mM l-NAME supplemented as previously described^58^. Flies were collected after eclosion and placed on L-NAME-supplemented food or normal food as a control. Flies were maintained at 29 °C and were transferred to fresh food every 3 days. The sample size included a minimum of ten fly brains per each genotype.

### Brain RNA isolation and quantitative PCR

RNA isolation and quantitative PCR was performed as previously described^23^. We isolated total RNA from 30–40 fly heads for each condition tested. Hemizygous males only were used for X chromosomal mutations. We used heterozygous females to evaluate transcript levels in Supplementary Fig. 6. The RNA extraction was carried out using Trizol (Invitrogen) followed by phenol-chloroform as per the manufacturer’s instructions. The total RNA for each sample was cleaned using the NEB RNA Cleanup kit (NEB T2030) according to the manufacturer’s instructions. We performed the qRT-PCR experiments using the Sybergreen Powerup master mix (Applied Biosystems) and the ABI7300 Real-Time Thermocycler. For each experiment, we performed three biological replicates per genotype and time point investigated. From this, we were able to obtain the average CT value for each biological replicate. Actin 5c was our internal control^81^. The fold change in expression relative to wildtype was found using the 2^(−ΔΔCT)^ method for both mutants and RNAi knockdown experiments. The following primers were used as previously described:^54^

Actin 5c Forward primer 5′-CGAAGAAGTTGCTGCTCTGGTTGT-3′

Actin 5c Reverse primer 5′-GGACGTCCCACAATCGATGGGAAG-3′

Attacin C Forward primer 5′-CTGCACTGGACTACTCCCACATCA-3′, Attacin C Reverse primer 5′-CGATCCTGCGACTGCCAAAGATTG-3′, Cecropin A1 Forward primer 5′-CATTGGACAATCGGAAGCTGGGTG-3′, Cecropin A1 Reverse primer 5′-TAATCATCGTGGTCAACCTCGGGC-3′, Defensin Forward primer 5′-CCAGAGGATCATGTCCTGGTGCAT-3′ Defensin Reverse primer 5′-ACTTGGAGAGTAGGTCGCATGTGG-3′, Diptericin B Forward primer 5′-AGGATTCGATCTGAGCCTCAACGG-3′, Diptericin B Reverse primer 5′-TGAAGGTATACACTCCACCGGCTC-3′, Drosomycin Forward primer 5′-AGTACTTGTTCGCCCTCTTCGCTG-3′, Drosomycin Reverse primer 5′-CCTTGTATCTTCCGGACAGGCAGT-3′, Metchnikowin Forward primer 5′-CATCAATCAATTCCCGCCACCGAG-3′, Metchnikowin Reverse primer 5′-AAATGGGTCCCTGGTGACGATGAG-3′.

### Nitric oxide signaling dye

The use of DAR 4 M AM nitric oxide signaling dye was adapted from ref. ^57^. Brains were dissected in 1X PBS. Each sample was then incubated in 10 μM DAR 4 M AM at room temperature (RT) in the dark for 1 h. Brains were then fixed for 20 min using 4% paraformaldehyde at RT. Samples were washed using 0.3% PBST four times at 5 min per wash. Brains were mounted using Vectashield media and immediately imaged. The sample size consisted of a minimum of 13 brains per genotype. Once imaged slides were stored at −20 °C.

### Statistical analysis

Statistical analysis for the data were produced using Brown–Forsythe and Welch ANOVA tests with post hoc Games–Howell’s multiple comparisons or a Student’s *t*-test where appropriate using Graphpad Prism (Graphpad Software, Inc.).

### Reporting summary

Further information on research design is available in the Nature Research Reporting Summary linked to this article.

## Supplementary information

Supplementary Material

Reporting Summary

## Data Availability

The authors affirm that the conclusions of the article are present within the article, figures, and tables.
