# Supplementary material for: Vexed mutations promote degeneration of dopaminergic neurons through excessive activation of the innate immune response
Source: NPJ Parkinsons Dis. 2022 Nov 2;8:147. doi: 10.1038/s41531-022-00417-5 (PMC9630459; doi:10.1038/s41531-022-00417-5)
Supplement: Supplementary file 1 — Supplementary Material [file 41531_2022_417_MOESM1_ESM.pdf]

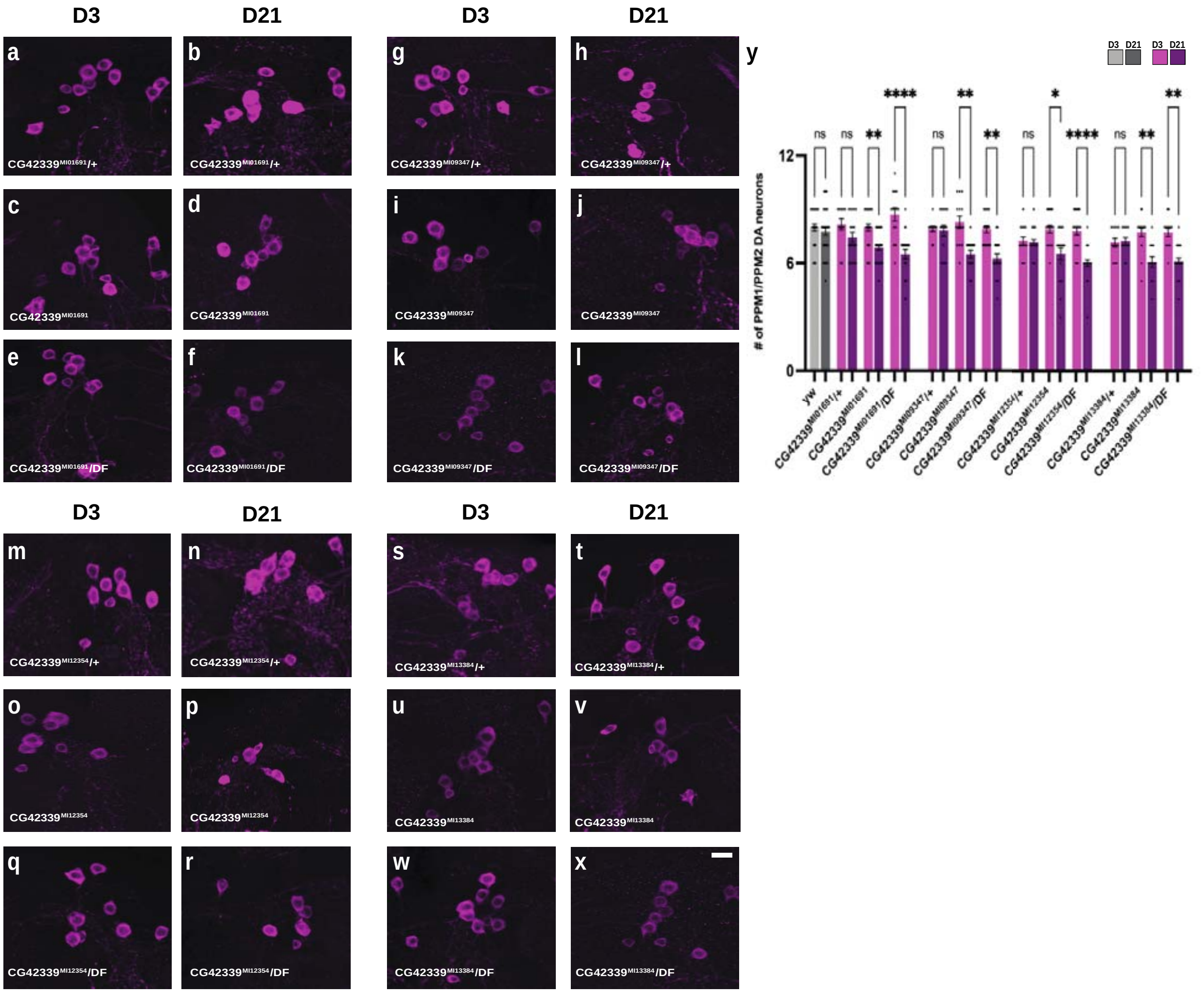

Supplementary Figure 1. Progressive loss of PPM1/2 neurons in *CG42339* mutants

(A-Y) Progressive loss of PPM1/2 neurons stained with anti-Tyrosine Hydroxylase (magenta). Neuronal loss was assessed in $CG42339^{MI01691}$ (A-F) $CG42339^{MI09347}$(G-L) $CG42339^{MI12354}$ (M-R) and $CG42339^{MI13384}$ (S-X). Each allele was assessed in both heterozygous and homozygous conditions, as well as over the deficiency *BSC540* that spans this region. Individual data points are shown with black dots. Images were taken at 20x magnification with Z stack slice interval 1.00 μm zoomed to 3.5x. Error bars represent the S.E.M. ****$p<0.0001$;***$p<0.001$;**$p<0.01$;

*p<0.05; n.s., not significant using Brown-Forsythe and Welch ANOVA tests with Post hoc Games-Howell's multiple comparisons. Scale bar in X is 12 μm for A-X.

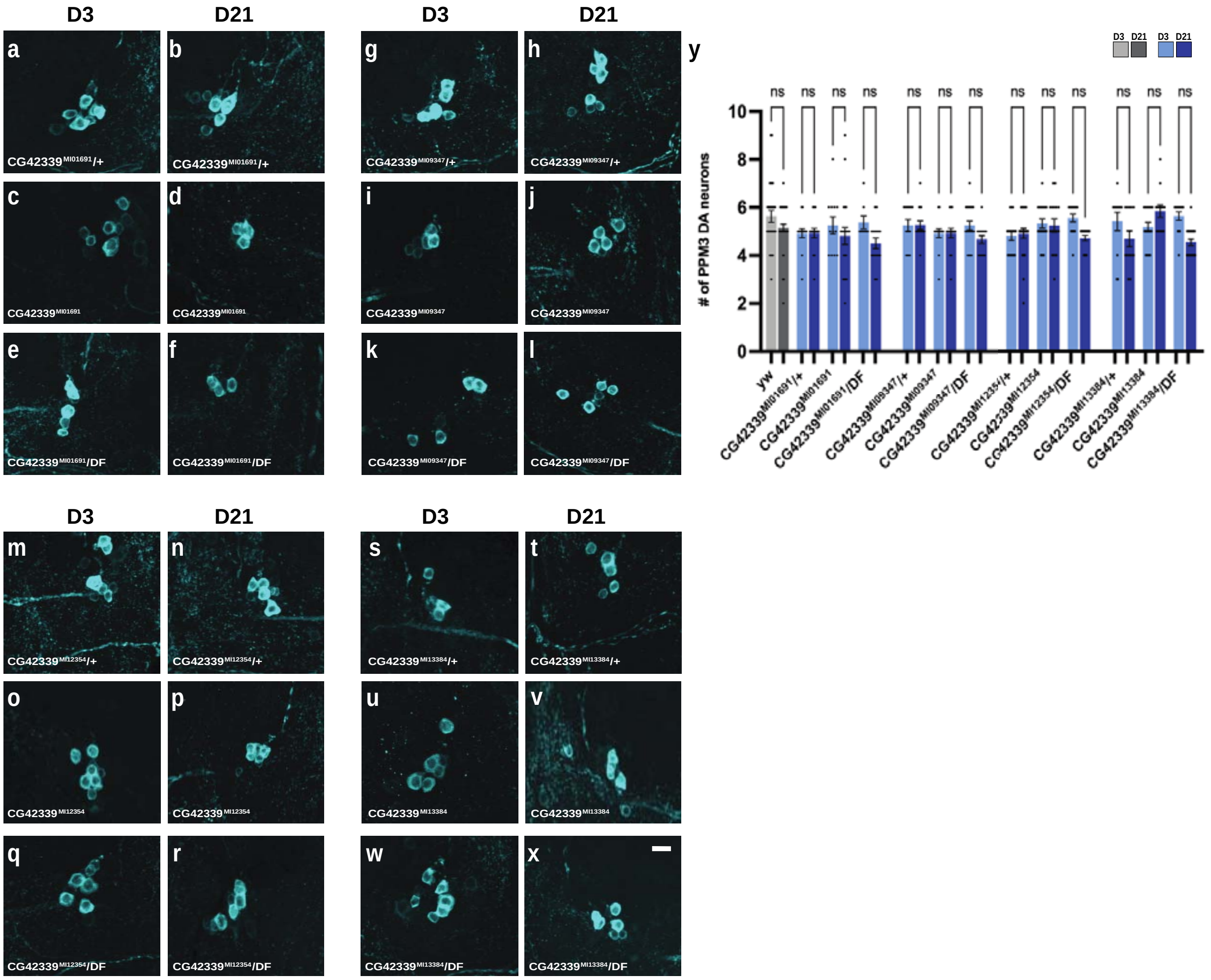

Supplementary Figure 2. Viability of PPM3 neurons in *CG42339* mutants

(A-Y) Viability of PPM3 neurons stained with anti-Tyrosine Hydroxylase (cyan). PPM3 neurons were assessed in *CG42339^MI01691^* (A-F) *CG42339^MI09347^*(G-L) *CG42339^MI12354^* (M-R) and *CG42339^MI13384^* (S-X). Each allele was assessed in both heterozygous and homozygous conditions, as well as over the deficiency *BSC540* that spans this region. Images were taken at 20x magnification with Z stack slice interval 1.00 µm zoomed to 3.5x. Individual data points are shown with black dots. Error bars represent the S.E.M. n.s., not significant using Brown-Forsythe and Welch ANOVA tests with Post hoc Games-Howell's multiple comparisons. Scale bar in X is 12 µm for A-X.

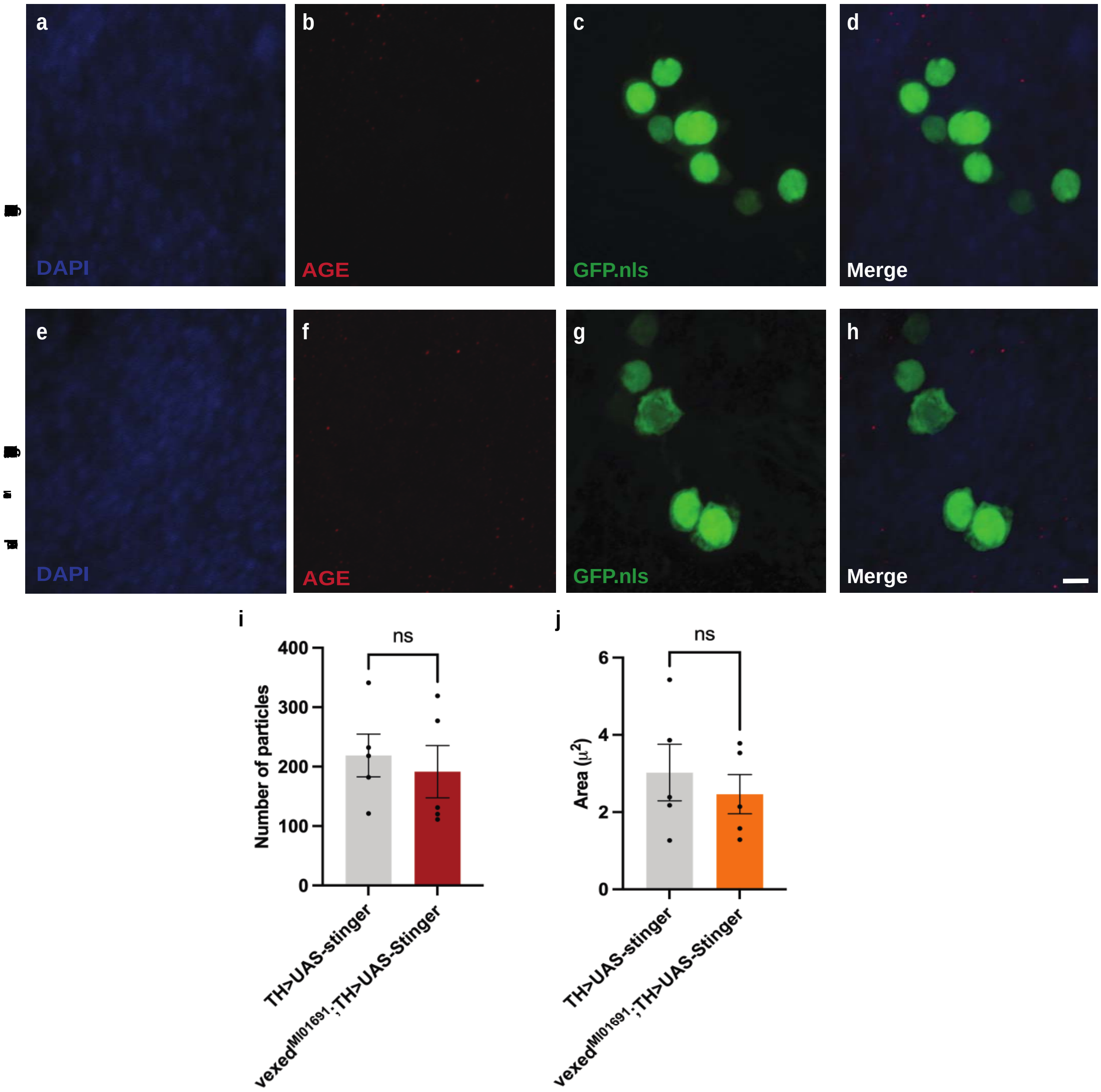

Supplementary Figure 3. Levels of Advanced Glycation End Products near PPM3 neurons

(A-H) While using DA-neuron specific expression of a nuclear-localized GFP (*UAS-Stinger*) in a wildtype (A-D) or *CG42339* mutant (E-H) background, we did not detect a significant amount of

AGE accumulation near the PPM3 neurons. Images were taken at 63x magnification with Z stack slice interval 0.88 μm zoomed to 3.5x. **(I)** Average number of AGE particles found within an area of 1500 μm^2 in both wildtype and *CG42339* mutant brains. **(J)** Average area occupied by AGE staining within an area of 1500 μm^2 in both wildtype and *CG42339* mutant brains. Individual data points are shown with black dots. Error bars represent the S.E.M. n.s. not significant; using a Student's T-test. Scale bar in H is 5 μm for A-H.

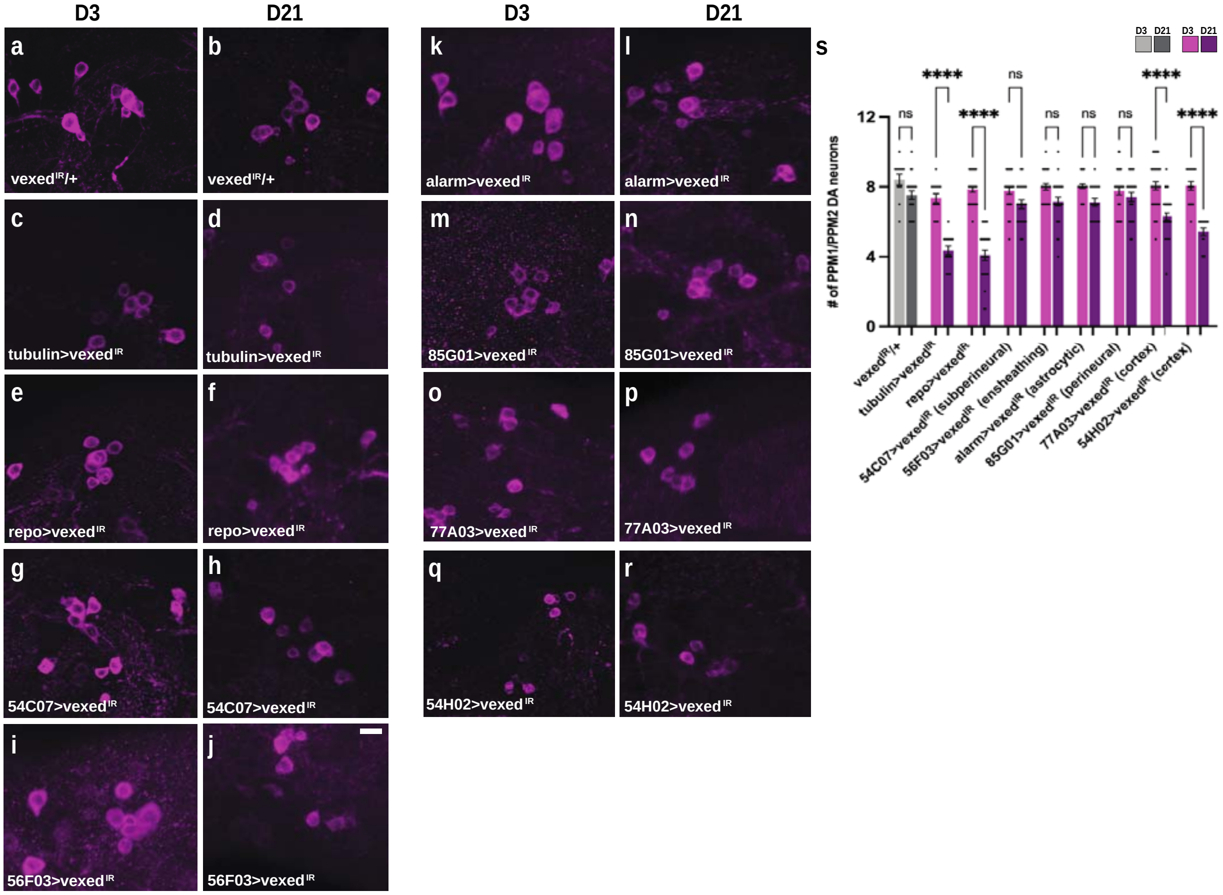

Supplementary Figure 4. Loss of PPM1/2 neurons upon knockdown of *vexed* in cortex glia

(A-S) Clusters of PPM1/2 neurons stained with anti-Tyrosine Hydroxylase (magenta) upon tissue-specific knockdown of *vexed*. Comparison of controls (A,B) to ubiquitous knockdown (C,D), and knockdown in all glia (E,F), subperineural glia (G,H), ensheathing glia (I,J), astrocytic glia (K,L), perineural glia (M,N), and cortex glia (O-R). Images were taken at 20x magnification with Z stack slice interval 1.00 μm zoomed to 3.5x. Individual data points are shown with black dots. Error bars represent the S.E.M. ****$p<0.0001$; n.s., not significant using Brown-Forsythe and Welch ANOVA tests with Post hoc Games-Howell's multiple comparisons. Scale bar in J is 12 μm for A-R.

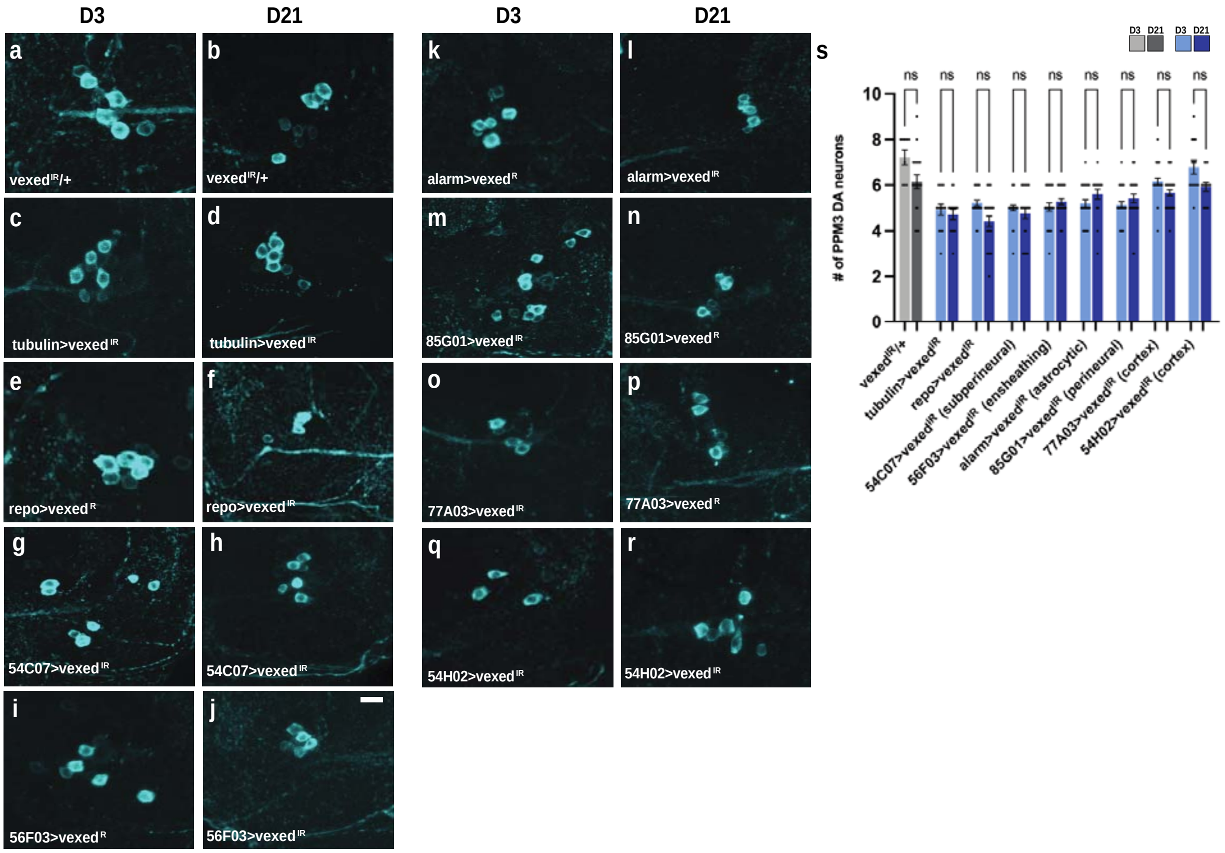

Supplementary Figure 5. Viability of PPM3 neurons upon knockdown of *vexed* in cortex glia

(A-S) Clusters of PPM3 neurons stained with anti-Tyrosine Hydroxylase (cyan) upon tissue-specific knockdown of *vexed*. Comparison of controls (A,B) to ubiquitous knockdown (C,D), and knockdown in all glia (E,F), subperineural glia (G,H), ensheathing glia (I,J), astrocytic glia (K,L), perineural glia (M,N), and cortex glia (O-R). Images were taken at 20x magnification with Z stack slice interval 1.00 µm zoomed to 3.5x. Individual data points are shown with black dots. Error bars represent the S.E.M. n.s., not significant using Brown-Forsythe and Welch ANOVA tests with Post hoc Games-Howell's multiple comparisons. Scale bar in J is 12 µm for A-R.

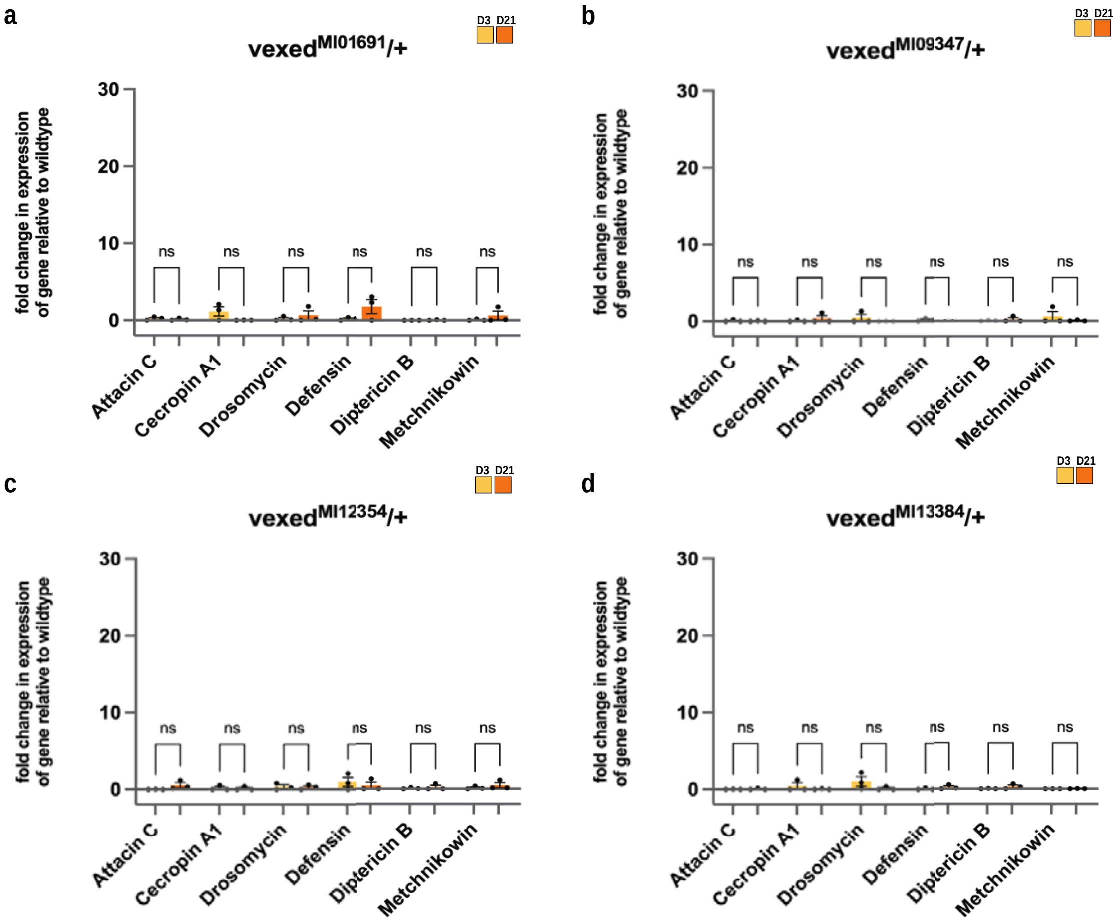

Supplementary Figure 6. Transcript levels of antimicrobial peptides for heterozygous *vexed* mutants

(A-D) Assessment of *vexed* heterozygous mutant alleles using qPCR to analyze transcript levels of antimicrobial peptides at day 3 compared to day 21 relative to wildtype expression. Error bars represent the S.E.M. n.s., not significant using Brown-Forsythe and Welch ANOVA tests with Post hoc Games-Howell's multiple comparisons.

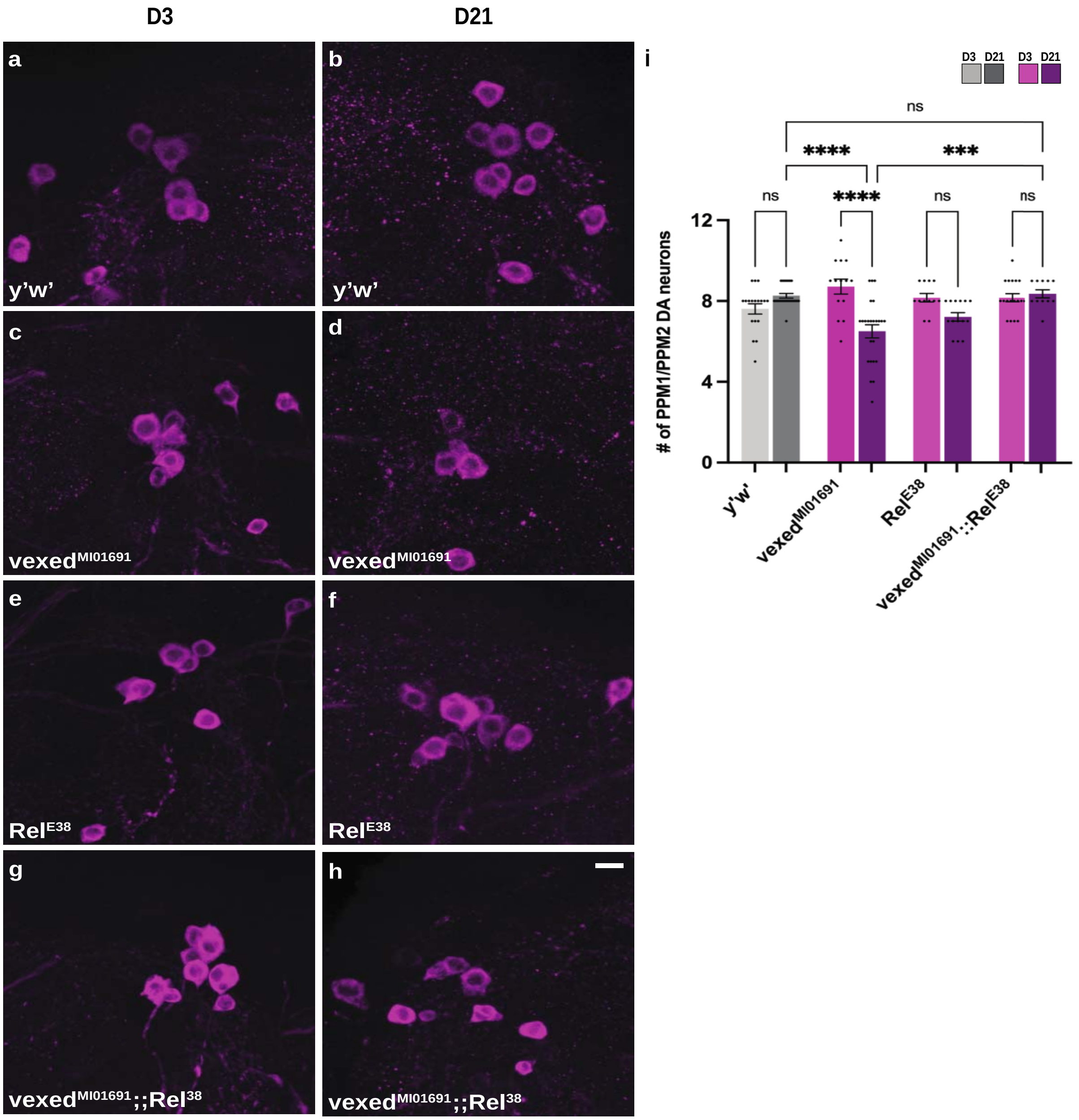

Supplementary Figure 7. Rescue of PPM1/2 neurons in *vexed* ; *relish* double mutants

(A-I) Measurement of PPLM1/2 neuron viability in wildtype controls (A,B), *vexed* mutants (C,D), *relish* mutants (E,F), and flies with mutations in both *vexed* and *relish* (G,H). Images were taken

at 20x magnification with Z stack slice interval 1.00 μm zoomed to 3.5x. Individual data points in each graph are shown with black dots. Error bars represent the S.E.M. ****$p<0.0001$; ***$p<0.001$; n.s., not significant using Brown-Forsythe and Welch ANOVA tests with Post hoc Games-Howell's multiple comparisons. Scale bar in H is 12 μm for A-H.

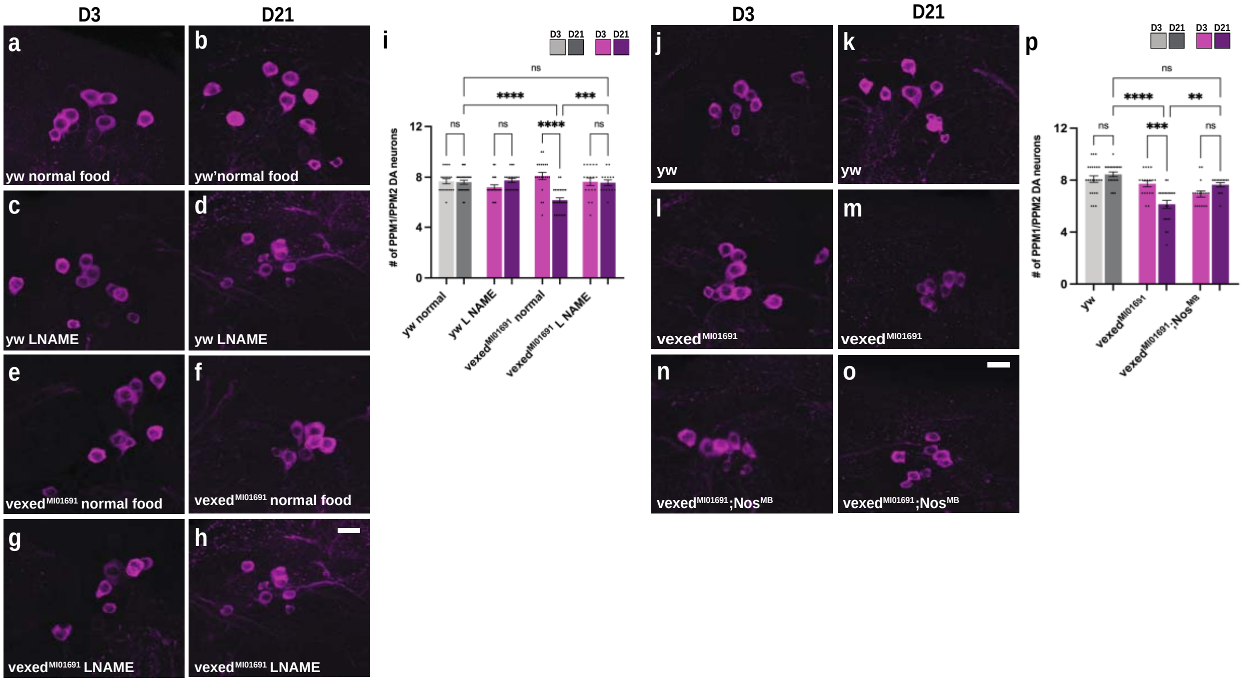

Supplementary Figure 8. Rescue of PPM1/2 neurons by pharmacological or genetic reduction in nitric oxide signaling

(A-I) Viability of PPM1/2 neurons upon pharmacological inhibiting nitric oxide signaling with L-NAME. Conditions tested were wildtype controls raised on standard (A,B) or L-NAME-supplemented food (C,D) and vexed mutants raised on standard (E,F) or L-NAME-supplemented food (G,H). **(J-P)** Viability of PPM1/2 neurons upon genetically inhibiting nitric oxide signaling using a *nos* mutant allele. Conditions tested were wildtype controls (J,K), *vexed* mutants (L,M), and flies with mutations in both *vexed* and *nos* (N,O). Images were taken at 20x magnification with Z stack slice interval 1.00 µm zoomed to 3.5x. Individual data points in each graph are shown with black dots. Error bars represent the S.E.M. ****p<0.0001; ***p<0.001; **p<0.01; n.s., not

significant using Brown-Forsythe and Welch ANOVA tests with Post hoc Games-Howell's multiple comparisons. Scale bar in H is 12 μm for A-H. Scale bar in O is 12 μm for J-O.
